# Toward Artificial Peptide Nanocapsules

**DOI:** 10.1007/s40820-024-01443-z

**Published:** 2024-05-29

**Authors:** Yuan Wang, Bing Chen

**Affiliations:** 1 Laboratory for Biomaterials and Drug Delivery, Department of Anesthesiology, Division of Critical Care Medicine, Boston Children’s Hospital, Harvard Medical School, Boston, MA 02115 USA; 2 College of Electronic and Optical Engineering and College of Flexible Electronics (Future Technology), Nanjing University of Posts and Telecommunications, Nanjing, 210023 People’s Republic of China

**Keywords:** Peptide nanocapsules, Self-assembly, Drug delivery, Nanomedicine

## Abstract

- The formation of peptide nanocapsules is facilitated by a gradient interface, where the differential solvent concentration drives the peptides to preferentially localize and assemble.
- The peptide nanocapsules, characterized by their hollow structures, demonstrated potential as carriers for targeted drug delivery.

## Introduction

Peptide nanocapsules are a type of nanoscale delivery system that encapsulates active substances within a shell composed of peptides, leveraging the unique properties of peptides such as biocompatibility and biodegradability [1]. Historically, the development of peptide nanocapsules was inspired primordially by the natural biological processes. For instance, cellular motility is largely regulated by the cytoskeletal proteins, which reversibly assemble to facilitate rhythmed extension and contraction of cells. Besides, viruses are simply composed of genetic materials including deoxyribonucleic acid (DNA) or ribonucleic acid (RNA) and protein capsids, capable of using capsids to protect and deliver their genetic materials into host cells. Researchers have mimicked these natural systems to design synthetic nanocapsules that can similarly load up cargoes and deliver therapeutic agents. In the laboratory, peptide nanocapsules are typically formed through self-assembly processes, where peptides are designed to spontaneously organize into a desired structure using techniques such as micro-emulsion, copolymerization, solvent-switching, pH change, and ionic strength adjustment to trigger assembly [2]. In addition, advanced molecular engineering techniques also enable the use of hybrid materials that combine peptides with other substances like polymers or lipids to enhance functional properties [3]. However, compared to the natural biological processes in which biological entities efficiently utilize gradients in chemical composition and physical properties to assemble intricate structures, replicating such precise control in synthetic systems poses a significant challenge.

**Fig. 1:**
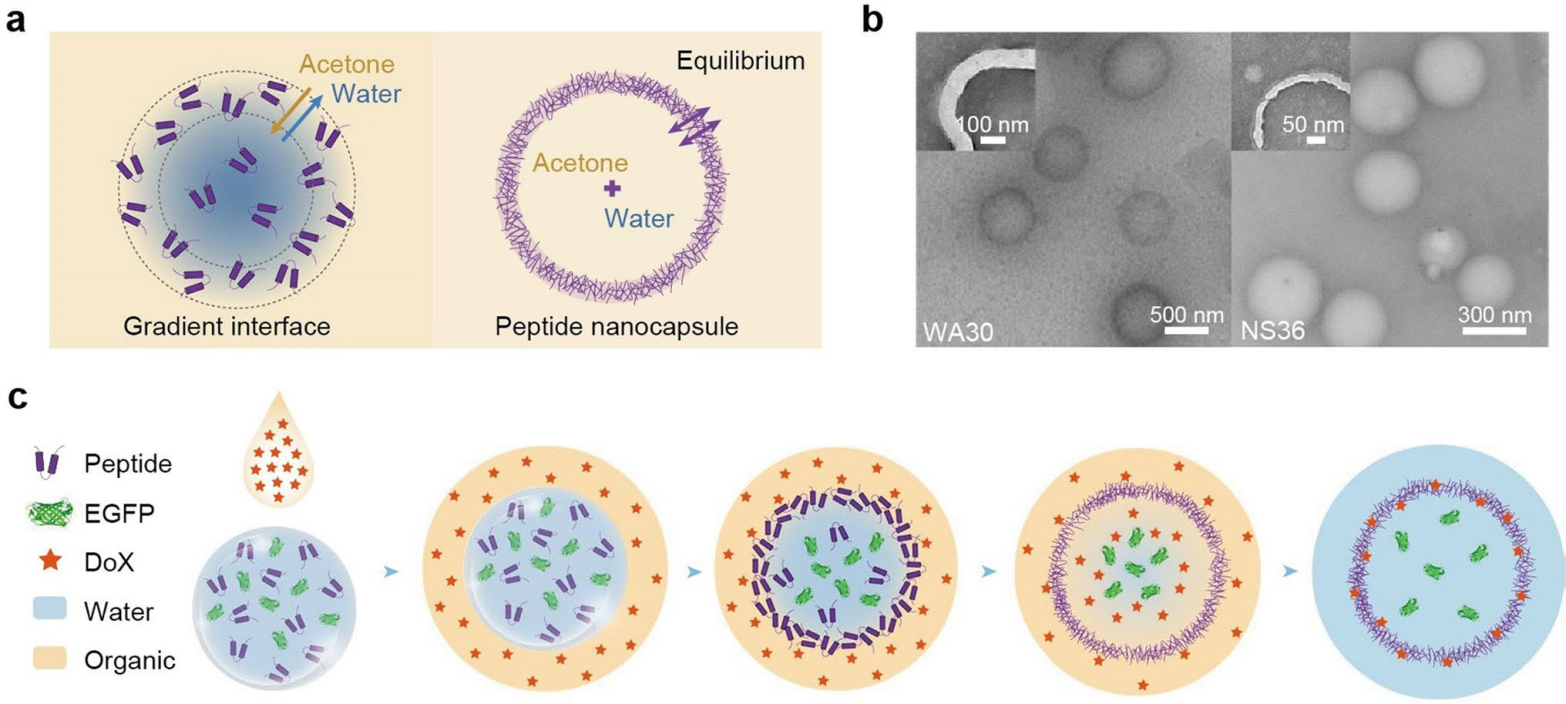
**a** Schematic of the solvent gradient-mediated assembly of ICP nanocapsules. **b** Transmission electron microscopy images of the ICP nanocapsules: WA30 (left panel) and NS36 (right panel) nanocapsules, respectively. **c** Schematic of encapsulating DoX and EGFP into the ICP nanocapsules for nanomedicine. Figures adapted from Ref. [4], Copyright©2024, The Author(s), under exclusive licence to Springer Nature Limited

Recent advancements in nanotechnology and biomimetics have opened avenues for designing complex structures capable of mimicking biological systems. Now writing in *Nature Nanotechnology*, Yu and colleagues from Nanyang Technological University. represented a significant breakthrough in the field of the self-assembly of insect cuticle peptides (ICPs) into peptide nanocapsules using a simple solvent-exchange technique, termed nanoprecipitation (Fig. 1a) [4]. This process exploits the natural tendency of insect cuticle peptides (such as WA30 and NS36) to assemble into hollow nanocapsules when exposed to a concentration gradient formed by mixing water and acetone (Fig. 1b). The study highlights the formation of peptide nanocapsules facilitated by a gradient interface, where the differential solvent concentration drives the peptides to preferentially localize and assemble. Unlike traditional methods requiring external scaffolds or templates, this study harnesses the intrinsic properties of peptides to direct self-assembly, thereby simplifying the synthesis of functional nanomaterials. The research also showcases how varying the solvent composition can influence the structural attributes of the nanocapsules, such as wall thickness and diameter, providing a tunable platform for biomedical applications. Given the hollow nature, this research underscores the potential of gradient-mediated self-assembly as a powerful strategy for fabricating biomimetic nanomaterials as carriers for targeted drug delivery (Fig. 1c). The ability of these peptide nanocapsules to encapsulate cargo with high loading efficiency (> 85%, for green fluorescent protein, DNA, RNA, β-galactosidase, bovine serum albumin, Smac and doxorubicin hydrochloride) and release drugs in a controlled manner offers promising avenues for enhancing the efficacy of therapeutic agents, potentially revolutionizing how treatments are delivered at the cellular level. More importantly, the simplicity and efficiency of the method pave the way for its application in developing drug delivery systems that mimic biological precision and complexity.

It is supposed that the successful implementation of this solvent gradient technique for peptide self-assembly opens several research directions, extending far beyond the field of materials synthesis. Future investigations could focus on understanding the specific interactions and conditions that favor certain structural configurations, the comparative effectiveness of these biomimetic structures against existing drug delivery platforms, and the scalability of this method for clinical settings.

## Mechanism Clarification

It is pivotal to further understand how peptides interact with different solvents (like water and acetone in the study) and how they react to changes in temperature, pH, or concentration gradients. Under some circumstances, certain structural configurations could lead to the development of customizable drug delivery systems that have the characteristic of reversible stimulus response [5]. Moreover, different medical conditions require different strategies for effective treatment. Understanding the aforementioned interactions and conditions allows scientists to design drug delivery systems that can be customized. For example, cancer treatments might need targeted delivery (e.g., doxorubicin) to tumor cells without affecting healthy tissue, whereas chronic pains require a sustained release of anesthetics (e.g., tetrodotoxin) for long-acting anesthesia. By understanding how to control peptide self-assembly into nanocapsules, researchers can create systems that are optimized for delivering drugs in ways that are most effective for treating.

## Unique Advantages

It is important to evaluate how well these new, biologically inspired structures perform in comparison to currently used drug delivery systems to administer therapeutic agents to patients, such as liposomes, polymeric nanoparticles, dendrimers, micelles, and conventional drug delivery systems like intramuscular injection. The main indicators include efficacy, safety, stability, and controllability. The goal of this comparison is to determine whether these new biomimetic nanocapsules offer significant advantages over traditional methods, such as improved treatment outcomes, reduced side effects, or more precise drug delivery, to replace or complement existing drug delivery systems in clinical settings. The integration of computational models and experimental biology could accelerate the design of next-generation nanocapsules with enhanced functional properties, such as targeted delivery and triggered release.

## Clinical Prospect

The scalability of this peptide nanocapsule technique for clinical settings is also vital. Often, methods developed in a research setting must undergo significant modifications to be practical for real-world clinical applications. Scalability involves adapting these small-scale, experimental methods to the larger-scale requirements of medical practice. There is no doubt that these peptide nanocapsules must be economically viable when scaled up. Specifically, the costs associated with larger-scale production, distribution, and administration need to be balanced by the benefits they offer, such as improved patient outcomes or reduced overall treatment costs. As the production scales up, it is vital to maintain the quality and effectiveness of the peptide nanocapsules. This includes ensuring that the nanocapsules remain stable, retain their drug-carrying capacity, and consistently release their payload in the intended manner.

## Conclusions

In conclusion, the study by Yu and colleagues not only advances our understanding of the fundamental principles of peptide self-assembly but also highlights the practical implications of these processes in nanotechnology and nanomedicine. The innovative approach of using solvent concentration gradients to facilitate the self-assembly of functional nanomaterials offers a promising pathway toward the development of sophisticated, bio-inspired systems with potential applications in various fields, including diagnostics, therapeutics, regenerative medicine and beyond.
